# Why don't physicians adhere to guideline recommendations in practice? An analysis of barriers among Dutch general practitioners

**DOI:** 10.1186/1748-5908-4-54

**Published:** 2009-08-12

**Authors:** Marjolein Lugtenberg, Judith M Zegers-van Schaick, Gert P Westert, Jako S Burgers

**Affiliations:** 1 Scientific Centre for Transformation in Care and Welfare (Tranzo), Tilburg University, PO Box 90153, 5000 LE Tilburg, The Netherlands; 2 Amphia hospital, Department of Cardiology, PO Box 90158, 4800 RK, Breda, The Netherlands; 3 National Institute for Public Health and the Environment (RIVM), PO Box 1, 3720 BA Bilthoven, The Netherlands; 4 Scientific Institute for Quality of Healthcare (IQ Healthcare), University Medical Centre St. Radboud, PO Box 9101, 6500 HB Nijmegen, The Netherlands

## Abstract

**Background:**

Despite wide distribution and promotion of clinical practice guidelines, adherence among Dutch general practitioners (GPs) is not optimal. To improve adherence to guidelines, an analysis of barriers to implementation is advocated. Because different recommendations within a guideline can have different barriers, in this study we focus on key recommendations rather than guidelines as a whole, and explore the barriers to implementation perceived by Dutch GPs.

**Methods:**

A qualitative study using six focus groups was conducted, in which 30 GPs participated, with an average of seven per session. Fifty-six key recommendations were derived from twelve national guidelines. In each focus group, barriers to the implementation of the key recommendations of two clinical practice guidelines were discussed. Focus group discussions were audiotaped and transcribed verbatim. Data was analysed by using an existing framework of barriers.

**Results:**

The barriers varied largely within guidelines, with each key recommendation having a unique pattern of barriers. The most perceived barriers were lack of agreement with the recommendations due to lack of applicability or lack of evidence (68% of key recommendations), environmental factors such as organisational constraints (52%), lack of knowledge regarding the guideline recommendations (46%), and guideline factors such as unclear or ambiguous guideline recommendations (43%).

**Conclusion:**

Our study findings suggest a broad range of barriers. As the barriers largely differ within guidelines, tailored and barrier-driven implementation strategies focusing on key recommendations are needed to improve adherence in practice. In addition, guidelines should be more transparent concerning the underlying evidence and applicability, and further efforts are needed to address complex issues such as comorbidity in guidelines. Finally, it might be useful to include focus groups in continuing medical education as an innovative medium for guideline education and implementation.

## Background

Clinical practice guidelines are commonly regarded as useful tools for quality improvement [1]. However, their impact on clinical practice is not optimal. Several reviews have shown that guidelines have only been moderately effective in changing the process of care, and that there is much room for improvement [2-6]. For instance, general practitioners (GPs) in the Netherlands do not prescribe drugs according to the national guidelines in about one-third of cases, and this figure has stayed fairly constant during the last few years [7,8]. In addition, levels of adherence vary largely between practices and between diagnoses [7-9].

To improve adherence to guidelines in practice, an analysis of barriers to implementation of guidelines among target users is advocated [10,11]. A large number of potential barriers have been identified operating at different levels, such as the level of the practitioner, the level of the patient, the organisational context, and the social and cultural context [10-14]. A recently conducted review and synthesis of qualitative studies [15] identified six themes of barriers to the implementation of guidelines among GPs: the content of the guidelines, the format of the guidelines, GPs individual experience, preserving the doctor-patient relationship, professional responsibility, and practical issues.

Few studies have focussed on a set of guidelines considering the variety of barriers that should be addressed to improve guideline adherence [12]. In addition, guideline studies often focus on barriers regarding the guideline as a whole, rather than on barriers operating at the level of the individual recommendations within the guidelines [16-19]. As different recommendations within the same guideline can have different barriers, it might be more useful to focus on barriers of individual recommendations to optimize the strategies needed for implementation of guidelines in practice.

The aim of this study was to identify the perceived barriers towards the use of national guidelines for general practice by focusing on the key recommendations within the guidelines. By analysing multiple key recommendations from a set of guidelines, we aim to identify which barriers occur most frequently across the selection. These findings may be useful for guideline developers as well as for professional organisations in designing tailored implementation strategies.

## Methods

### Setting

The Dutch College of General Practitioners (NHG) has developed a set of more than 80 national guidelines that cover the majority of conditions and diseases seen in general practice [20]. The guidelines have been developed according to the principles of evidence-based medicine, formulating recommendations based on the best available evidence [21]. Along with the development of guidelines, NHG also puts considerable effort into promoting the use of these guidelines among the target group. They select key recommendations within each guideline, provide a two-page summary, and supply tools for application, such as electronic decision tools, patient information leaflets, and educational materials. In addition, continuing medical education (CME) for GPs in the Netherlands is only accredited if it is based on this set of nationally endorsed guidelines.

### Study design

Six two-hour focus group sessions were conducted in which twelve NHG guidelines were discussed. Focus groups have proven to be a useful method of providing in-depth information and exploring cognitions and motivations underlying behaviour [22-25]. This is particularly useful when behaviour change is needed. The focus groups enabled us to identify the most relevant barriers perceived by GPs in applying guidelines in practice.

### Selection of clinical guidelines

An expert panel of GPs (n = 16) was asked to help selecting the guidelines for our study. The panel was recruited by the organisation responsible for CME for GPs in the Southwestern part of the Netherlands (Stichting KOEL) [26]. We provided an overview of the NHG guidelines published since 2003 and asked the panel members for each guideline about the relevance of studying the effects of the guideline on quality of care and the potential improvement of quality of care as a result of implementing the guideline. In addition, they were asked to select five guidelines that should have high priority as part of a guideline implementation study.

The panel suggested nineteen guidelines having high priority. From these nineteen, we selected twelve guidelines according to the equal distribution among prevalence and type of diseases, and the measurability of quality improvement on patient outcomes (Table 1). Fifty-six key recommendations were abstracted from the twelve guidelines (Additional File 1, in Dutch).

**Table 1:** Selected guidelines

Guideline	Number of key recommendations	Year of publication
Asthma among children	7	2006
Atrial fibrillation	5	2003
Cardiovascular risk management	7	2006
Cerebrovascular accident	5	2004
Depressive disorder	5	2003
Eye inflammation ('red eye')	3	2006
Rhinosinusitis	2	2005
Sexually transmitted diseases	4	2004
Sleeping disorder	7	2005
Thyroid disorders	3	2006
Transient ischemic attack	3	2004
Urinary tract infections	5	2005

### Selection of participants

GPs were recruited by Stichting KOEL through advertisement in their electronic newsletter and website. They could register for more than one focus group session and were offered CME accreditation (two hours per session). All 34 GPs that had registered for one or more focus group sessions were invited and 30 of them (88%) participated in the sessions (range, 5 to 13). Nine of them participated in two sessions and one in all six sessions. One-half of the participants were male, and most of them were between 45 and 54 years of age (37%), practiced in a group setting (45%), and worked in a rural area or small town (39%). Compared to the total population of Dutch GPs [27], participants working in group practices and in towns or small cities were slightly overrepresented.

### Focus groups sessions

The participants received a copy of the key recommendations of the guidelines one week in advance. In each focus group session, the GPs had a semi-structured discussion about the perceived barriers to the implementation of the key recommendations of two guidelines. The sessions were chaired by a GP with at least 15 years of experience in general practice and guideline development (JB), and co-chaired by a health services researcher (ML). A topic guide with open-ended questions was used to structure the discussion. The six sessions were held at Stichting KOEL from March to June 2008 and were audiotaped.

### Data analysis and synthesis

The focus groups were transcribed verbatim. Two researchers (ML and JZ) independently studied the transcripts and classified the comments according to the framework of Cabana *et al*. [12]. In this framework, three main categories of barriers to following guidelines are distinguished: barriers related to knowledge, barriers related to attitude, and external barriers that are subdivided into several subcategories. For those comments that did not fit into the categories of the framework, additional types of barriers were formulated (Table 2).

**Table 2:** Perceived barriers* to the implementation of key recommendations from selected guidelines

Perceived barriers	Key recommendations (N = 56)		Clinical guidelines (N = 12)	
	
	N	%	N	%
Knowledge	26	46	10	83
Lack of knowledge	26	46	10	83
Lack of awareness/familiarity	26	46	10	83
Attitude	51	91	12	100
Lack of agreement with guideline recommendation	38	68	12	100
Interpretation/lack of evidence**	13	23	9	75
Lack of applicability	32	57	12	100
Lack of self-efficacy	11	20	8	67
Lack of outcome expectancy	17	30	10	83
Inertia of previous practice/lack of motivation	15	27	8	67
Behaviour	46	82	12	100
Patient factors	22	40	11	92
Patients preferences/demands	14	25	9	75
Patients ability/behaviour**	11	20	8	67
Guideline recommendation factors	24	43	11	92
Unclear/ambiguous**	18	32	11	92
Incomplete/not up to date**	8	14	4	33
Not easy to use/too complex**	3	5	3	25
Environmental factors	29	52	12	100
Lack of time/time pressure	7	13	5	42
Lack of resources/materials	7	13	5	42
Organisational constraints	20	36	11	92
Lack of reimbursement	2	4	2	17

* Barriers were classified according to the framework of Cabana *et al*. (1999) with some additional types of sub-barriers (**)

Additionally, we further divided organisational constraints into organisational constraints within the own organisation or practice (such as opening hours or insufficient number of personnel/staff), organisational constraints outside the organisation (such as policies in hospitals or out of hours services), and organisational constraints between organisations (such as communication and collaboration with other healthcare providers). Results of the two researchers were compared and discrepancies were discussed until consensus was reached. When necessary, a third researcher (JB or GW) was consulted.

In the synthesis of the data, the key recommendation is the unit of analysis. For each barrier in our model, we calculated the number and percentage of key recommendations to which the barrier applied.

## Results

### Perceived barriers

Barriers related to attitude were perceived for 91% of the key recommendations; behaviour-related barriers and knowledge-related barriers were perceived for 82% and 46% of the key recommendations respectively (Table 2).

Within these three main categories, the most perceived barriers were lack of agreement with guideline recommendations (applicable to 68% of the key recommendations), followed by environmental factors (52%), lack of knowledge of the guideline recommendations (46%), and guideline recommendation factors (43%).

Table 3 presents the perceived types of barriers per guideline. In the following sections, the perceived barriers are discussed according to the main categories of barriers: knowledge, attitude and behaviour.

**Table 3:** Perceived barriers to the implementation of key recommendations per guideline

**Clinical practice guideline** (Number of key recommendations)	Knowledge	Atttitude				Behaviour		
	Lack of awareness/familiarity	Lack of agreement	Lack of self-efficacy	Lack of outcome expectancy	Inertia previous practice/lack of motivation	Patient factors	Guideline factors	Environmental factors
Asthma among children (7)	+	++	- -	+	- -	-	+	-
Atrial fibrillation (5)	- -	+	- -	- -	-	- -	+	++
Cardiovascular risk management (7)	- -	-	- -	- -	+	-	-	-
Cerebrovascular accident (5)	+	++	-	-	- -	- -	++	- -
Depressive disorder (5)	- -	+	- -	- -	- -	-	- -	- -
Eye inflammation (3)	-	+	-	-	+	+	- -	++
Rhinosinusitis (2)	- -	++	- -	++	- -	++	+	+
Sexually transmitted diseases (4)	++	+	+	-	+	+	++	+
Sleeping disorder (7)	+	-	- -	-	- -	-	- -	-
Thyroid disorder (3)	+	++	+	--	- -	+	-	++
Transient ischemic attack (3)	++	++	-	-	-	-	-	-
Urinary tract infections (5)	+	++	- -	-	-	- -	-	++
Mean 12 guidelines (4.7)	-	+	- -	-	-	-	-	+

-- barrier applicable to 0 to 25% of the key recommendations

- barrier applicable to 25 to 50% of the key recommendations

+ barrier applicable to 50 to 75% of the key recommendations

++ barrier applicable to 75 to 100% of the key recommendations

### Barriers related to knowledge

#### Lack of awareness/familiarity

GPs were generally aware of the guidelines, but did not know the specific content of 46% of the key recommendations (Table 2). GPs were mostly familiar with part of the key recommendation, but did not know, for instance, the recommended dosage of the drug (Appendix 1). Lack of awareness or familiarity was most relevant for the guidelines regarding transient ischemic attack and sexually transmitted diseases (Table 3).

### Barriers related to attitude

#### Lack of agreement with guideline recommendation

The most reported attitudinal barrier was a lack of agreement with the guideline recommendation (68%). This barrier was mostly related to a lack of applicability (57%) (Table 2). GPs felt that benefits often did not outweigh the harms, or that a recommendation was not applicable to a specific group of patients, such as patients with comorbidity (Appendix 2). Another reason why GPs did not agree with the recommendation was that they argued the evidence (or lack of evidence) underlying a recommendation (23%) (Appendix 2). Lack of agreement with guideline recommendations was a problem for all key recommendations in the guidelines for rhinosinusitis, thyroid disorders, transient ischemic attack, and urinary tract infection (Table 3).

#### Lack of self-efficacy

The lack of belief that one is capable of adequately performing the recommendation in practice was a barrier in 20% of the key recommendations. Reasons mentioned were a lack of skills, experience or training, or having more confidence in the expertise of other healthcare providers (Appendix 2). This type of barrier was most often mentioned for the key recommendations in the guidelines for thyroid disorders, and sexually transmitted diseases (Table 3).

#### Lack of outcome expectancy

In 30% of the key recommendations, GPs agreed with the content, but did not believe that applying the recommendation would result in better patient outcomes (Appendix 2). This was particularly a problem for the guidelines regarding rhinosinusitis, asthma among children, and sleeping disorder (Table 3).

#### Inertia of previous practice/lack of motivation

In 27% of the key recommendations, GPs were not sufficiently motivated to change, or felt that is was hard to overcome the inertia of previous practice due to habits and routines (Appendix 2). These barriers were most frequently mentioned for the guidelines regarding eye inflammation and cardiovascular risk management (Table 3).

### Barriers related to behaviour

#### Patient factors

Patient factors were mentioned as a barrier with respect to 40% of the key recommendations. In 25% of cases, GPs felt that patients' preferences did not match with the guideline recommendation (Table 2). Patient ability or behaviour was perceived as a barrier for 20% of the key recommendations, *e.g*., patients were not able to perform a required action accurately, or did not show up for follow-up (Appendix 3). Patient factors were most often reported as a barrier for the guidelines regarding rhinosinusitis, eye inflammation, and thyroid disorder (Table 3).

#### Guideline recommendation factors

In 43% of the key recommendations, factors related to the guideline were perceived as a barrier to implementation (Table 2). Recommendations were found to be unclear or confusing (32%), not covering all relevant information, or not being up to date (14%), or too complex or not easy to use in practice (5%) (Appendix 4). These types of barriers were most prominent for the guidelines regarding sexually transmitted diseases, cerebrovascular accident, and asthma among children (Table 3).

#### Environmental factors

Environmental factors were the most prominent barrier related to behaviour (52%) (Table 2). Particularly, organisational constraints were often reported as a barrier (36%). These constraints mostly referred to organisational constraints outside the organisation, such as logistic problems in out-of-hours services. Perceived constraints within the practice included communication and lack of education or skills among practice assistants. Constraints between organisations were unclear division of tasks and lack of collaboration with specialists in hospitals (Appendix 5). Other environmental barriers were lack of time (13%) and lack of resources (13%) (Appendix 5). Environmental barriers were relatively often perceived for the guidelines concerning eye inflammation, thyroid disorders, atrial fibrillation, and urinary tract infection (Table 3).

## Discussion

Our study revealed a broad spectrum of barriers that Dutch GPs perceive in applying the key recommendations of a set of nationally developed guidelines. Although the focus of the barriers differed across guidelines, each key recommendation had a unique combination of barriers. As a consequence, multiple interventions tailored to the specific barriers of the key recommendations are needed to improve the implementation of guidelines in practice.

The most prominent barrier was lack of agreement with guideline recommendations. GPs often disagreed with recommendations because they argued the underlying evidence provided or felt that it was not clear why they should apply them. In addition, they perceived some recommendations not being applicable due to heterogeneity of patient populations. Other studies also demonstrated that lack of applicability is an important barrier to guideline adherence, particularly to patients with comorbidity [18,28,29]. Evidence-based guidelines focus on patients with single diseases and often exclude complex patients, which limits the applicability in practice [30-33]. Further research and efforts are needed on methods to address comorbidity in guidelines in order to improve the applicability of guideline recommendations [31,32,34].

Environmental barriers, particularly organisational constraints, were the second most often perceived group of barriers to implementation. These constraints mostly referred to logistic problems within the own practice or within out-of-hours healthcare services. Moreover, lack of collaboration with other types of healthcare professionals was perceived as a barrier in our study, which is consistent with other studies [17,35-38]. Improvements can be made by better organising care and by improving multiprofessional collaboration. Standardisation of processes and procedures, and inter-professional agreements on referral and follow-up might be useful.

Dutch GPs are generally aware of the guidelines because they are a fundamental part of the postgraduate training and continuing medical education. This is a strong feature of the professionalisation of GPs that is rooted in the 1980s when the guideline program of the NHG started. Nevertheless, GPs did not know the content well for almost half of the key recommendations in the guidelines selected in our study. GPs might be confronted with too many guidelines, as each year eight to ten new guidelines or updated versions are produced. To improve knowledge on guidelines, it may be useful to regularly conduct sessions among GPs, because the participants in our study appreciated the focus group sessions and considered these as an innovative medium for guideline education and implementation. The effectiveness of interactive education with active involvement and participation has been demonstrated in other studies as well [39-41].

In our study, we found that guideline factors were a relevant barrier to implementation, which is consistent with previous studies [12,42]. GPs prefer short guideline recommendations that are easy to understand. The challenge is to produce simple and clear guideline recommendations that also address the complexity of problems seen in daily practice. Presenting guideline recommendations in multiple formats, such as algorithms, one or two page summaries, and electronic web-based versions with hyperlinks to more detailed information might serve the varying needs of physicians and patients [42,43].

We used an existing framework of barriers to guideline adherence from Cabana *et al*. [12], and explored whether it covered the full range of barriers perceived by GPs in our study. We suggest that lack of applicability should be a more prominent category, including different reasons such as that the benefits may not outweigh the harms or patients with comorbidity who need special attention. In addition, the external barriers could be extended with some subcategories, as presented in Table 2. Finally, organisational constraints could be subdivided into organisational constraints within the own organisation or practice, those outside the organisation and those between organisations. Other studies also suggested additions to the framework [44,45].

One of the strengths of our study is that we examined a large set of guidelines produced within one longstanding guideline program. Most qualitative studies have focused on a specific health topic, or studied only one or two guidelines [18,19,42,46,47], limiting the applicability of their findings. Secondly, we focused on barriers to key recommendations, rather than on barriers to guidelines as a whole. Our in-depth analysis of barriers provides detailed information on potential interventions needed to improve guideline adherence. This information can be used by professional groups or organisations, regionally and nationally, to develop multifaceted interventions, tailored to the individual recommendations in the guideline. For example, to improve the implementation of the guideline on urinary tract infections, it was suggested to develop local protocols for diagnosis in out-of-hours services, as the recommendation on diagnosis (*i.e*., the use of a dipslide method) did not apply well in these settings. Finally, the findings from our study may be useful for guideline developers in the process of updating the guidelines to raise the acceptance and implementability of the guideline recommendations.

Several limitations should be considered in interpreting our findings. First, we collected opinions from a small sample of GPs, with GPs working in group practices and in towns and small cities being slightly overrepresented [27]. However, the aim of our focus group study was to identify possible barriers qualitatively, rather than quantifying their relative importance among a representative group of GPs. Results from this study will be used as input for a survey to be conducted among a larger sample of GPs in order to quantify our findings. Secondly, we only included GPs and no other healthcare professionals in our focus group sessions. As some of the barriers were related to behaviour of the practice assistants or practice nurses, it might be useful to include these professions in focus group sessions as well.

## Conclusion

In conclusion, we identified a wide range of barriers that Dutch GPs face when using national guidelines. Using the focus group method proved to be an effective method to collect information on barriers. Results from this study help explaining why GPs do not adhere to guideline recommendations in practice, and provide useful suggestions for improving adherence. Our study also illustrated that lack of adherence to individual recommendations is related to multiple barriers. A detailed, in-depth analysis of barriers, as conducted in this study, offers opportunities for professional organisations to develop multiple, barrier driven, and tailored interventions to improve adherence in practice.

## Competing interests

The authors declare that they have no competing interests.

## Authors' contributions

ML drafted and revised the manuscript, has been involved in designing and conducting the focus groups study, and in analysing and interpreting the data. JZ has made substantial contributions in analysing the data. GW was involved in designing the study and critically revising the manuscript. JB supervised the study and has been involved in designing the study, conducting the focus group sessions and critically revising the manuscript. All authors have read and approved the final manuscript.

## Appendix 1

Examples of perceived barriers related to knowledge

LACK OF AWARENESS/FAMILIARITY

Guideline Sleeping disorder

'Can I be really honest with you? I have never read the guideline, never looked at it, never...'

**Guideline Cerebrovascular accident **(KR 2)

'I did not know about 160 mg acetylsalicylic acid for the course of two weeks... I always start with 80 mg in patients with stroke.'

## Appendix 2

Examples of perceived barriers related to attitude

LACK OF APPLICABILITY – benefits do not outweigh the harms

**Guideline urinary tract infection **(KR 4)

'I usually prescribe ciprofloxacin for the course of 10 days, because Augmentin is badly tolerated according to my experience.'

LACK OF APPLICABILITY – not applicable to patient population

**Guideline depressive disorder **(KR1)

'In practice, you never see patients with depression only or anxiety disorder only. Both often overlap. Then, the management plan is unclear.'

INTERPRETATION/LACK OF EVIDENCE – lack of evidence

**Guideline atrial fibrillation **(KR3)

'I only do thyroid gland testing. I do not understand the need for testing Hemoglobin and glucose in patients with atrial fibrillation. What's the evidence?'

LACK OF SELF-EFFICACY

**Guideline thyroid disorders **(KR 2)

'I do not have experience in treating hyperthyroid patients and only see a few of them per year. I think this is not sufficient to build up expertise.'

LACK OF OUTCOME EXPECTANCY

**Guideline sleeping disorder **(KR 6)

'.as GP in training, I was motivated to stop long term use of hypnotics in patients with a sleeping disorder. But now, people tell me: don't do it, it demands a lot of energy, without any predicted result. Then you start thinking: hands off, leave it.'

INERTIA OF PREVIOUS PRACTICE

**Guideline cardiovascular risk management **(KR 4)

'The new guideline recommends using systolic blood pressure...in monitoring drug treatment in patients with hypertension. However, I am used to monitor diastolic blood pressure...and then I feel guilty if I see someone with 150...I think that's a big change.'

## Appendix 3

Examples of perceived barriers related to behaviour: patient factors 

PATIENT FACTORS – Patient preferences and demands

**Guideline rhinosinusitis **(KR2)

'There is a tension between the recommendation and patient demands. Patients expect antibiotics. This sometimes causes friction...yes.'

PATIENT FACTORS – Patient ability and behaviour

**Guideline asthma among children **(KR2)

'Some children perform well in spirometry, but with a very large number the results are totally invalid. Well, with some children it is just not going to work.'

**Guideline cardiovascular risk management **(KR 7)

'Yes, I try to, but there are always patients that do not show up for follow-up. Always. Also with medication.'

## Appendix 4

Examples of perceived barriers related to behaviour: guideline recommendation factors

GUIDELINE RECOMMENDATION FACTORS – Confusing/not clear

**Guideline asthma among children **(KR1)

'I read the recommendation [on allergy testing in children younger than six years] five times, and I still did not understand it!'

GUIDELINE RECOMMENDATION FACTORS – Incomplete/not up to date

**Guideline cerebrovascular accident **(KR1)

'This recommendation is based on obsolete opinions. You cannot keep patients with stroke at home. All of them should be immediately admitted to hospital.'

## Appendix 5

Examples of perceived barriers related to behaviour: environmental factors

ENVIRONMENTAL FACTORS – Organisational constraints (outside organisation)

**Guideline urinary tract infection **(KR1)

'How to use a dipslide in out-of-hours services on Sunday? Then you need someone who reads the results on Monday. That is really bothersome.'

ENVIRONMENTAL FACTORS – Organisational constraints (within own practice)

**Guideline eye inflammation **(KR2)

'I would like to reduce antibiotic prescriptions in patients with red eye, but the practice assistant often deals with these patients who ask for a prescription by telephone. The bottleneck is mainly in prescriptions requested over the telephone. There is an important improvement to make there, yes! As the assistant thinks that at any time a prescription is necessary.'

ENVIRONMENTAL FACTORS – Organisational constraints (between organisations)

**Guideline cerebrovascular accident **(KR 4/5)

'It is unclear what the hospital arranges and what we need to do when stroke patients return to their homes. There should be a formal handoff between hospital and the GP.'

ENVIRONMENTAL FACTORS – Lack of time/time pressure

**Guideline cardiovascular risk management **(KR 1/2)

'It's great what we could offer in cardiovascular risk management, but it would need full weekdays to realize this in practice.'

ENVIRONMENTAL FACTORS – Lack of/unpractical resources/materials

**Guideline sexually transmitted diseases **(KR3)

'There are different media, which is unpractical in use...and the media used in cervix streams can only be shortly preserved.'

## Supplementary Material

Additional file 1 **Key recommendations of guidelines (in Dutch)**. Description of the 56 key recommendations from the twelve included national guidelines (in Dutch). Click here for file
