# Supplementary material for: Why don't physicians adhere to guideline recommendations in practice? An analysis of barriers among Dutch general practitioners
Source: Implement Sci. 2009 Aug 12;4:54. doi: 10.1186/1748-5908-4-54 (PMC2734568; doi:10.1186/1748-5908-4-54)
Supplement: Additional file 1 — Key recommendations of guidelines (in Dutch). Description of the 56 key recommendations from the twelve included national guidelines (in Dutch). [file 1748-5908-4-54-S1.doc]

**Additional file: Key recommendations of guidelines (Dutch)**

**1. Asthma among children/ Astma bij kinderen** (M24; 2006)

**Kernaanbeveling 1:**

Bij kinderen tot 6 jaar met aanwijzingen voor allergie of een allergische rhinitis en bij alle kinderen vanaf 6 jaar met klinische aanwijzingen voor astma (recidiverend optreden van kortademigheid of astma, al dan niet na inspanning) wordt aanbevolen screenend allergologisch onderzoek d.m.v. RAST-test op inhalatie-allergenen te verrichten.

**Kernaanbeveling 2:**

Bij kinderen vanaf 6 jaar is spirometrie zinvol bij twijfel aan of ter bevestiging van de diagnose astma.

**Kernaanbeveling 3**:

Bij kinderen wordt de diagnose astma **niet** gesteld als een kind alleen hoest en niet piept volgens de ouders, en de dokter hoort geen piepen bij lichamelijk onderzoek terwijl het kind wel klachten heeft. Hetzelfde geldt voor kinderen met klachten van benauwdheid bij inspanning zonder piepen of nachtelijk hoesten zonder piepen.

**Kernaanbeveling 4**:

Raad roken door het kind zelf, door ouders/ verzorgers of door anderen in de omgeving van het kind dringend af.

**Kernaanbeveling 5:**

Het voorschrijven van allergeenwerende matras- en kussenhoezen is alleen zinvol als op meerdere onderdelen sanerings­maatregelen worden ingezet.

**Kernaanbeveling 6:**

Bij kinderen tot en met 6 jaar met (verdenking) astma wordt een bèta-2-sympathicomimeticum voorgeschreven via een dosisaerosol met een inhalatiekamer en wordt het effect altijd na 1 tot 2 weken geëvalueerd. Bij kinderen ouder dan 6 jaar met astma dient een poederinhalator voorgeschreven te worden.

**Kernaanbeveling 7:**

Bij onvoldoende effect van bronchusverwijder (en goed medicatiegebruik) of bij frequente klachten (twee of meer dagen per week luchtwegklachten of twee of meer inhalaties met bronchusverwijder per dag gedurende 1 tot 2 weken nodig) is onderhoudsbehandeling met inhalatiecorticosteroïden de aanbevolen medicamenteuze therapie.

#### **2. Atrial fibrillation/ Atriumfibrilleren** (M79; 2003)

**Kernaanbeveling 1:**

Het hartritme dient onderzocht te worden bij de volgende klachten en bevindingen: hartkloppingen, duizeligheid in de zin van licht gevoel in het hoofd of het gevoel flauw te vallen, wegrakingen, een verminderde inspanningstolerantie, tekenen van hartfalen, een TIA of CVA.

**Kernaanbeveling 2:**

Bij iedere patiënt bij wie de bloeddruk wordt gemeten, wordt het ritme en de frequentie van de hartslag vastgelegd.

**Kernaanbeveling 3**:

Bij het vermoeden van atriumfibrilleren maakt de huisarts een ECG en laat laboratoriumonderzoek (Hb, glucose, TSH, evt. K en Kr) uitvoeren. De diagnose atriumfibrilleren wordt gesteld op basis van het ECG.

**Kernaanbeveling 4**:

Patiënten met atriumfibrilleren en vermoeden van een hartklepafwijking op grond van auscultatie van het hart of wanneer er onduidelijkheid bestaat over het bestaan van hartfalen, worden verwezen voor echodiagnostiek van het hart (naar cardioloog of diagnostisch centrum indien beschikbaar). Patiënten met atriumfibrilleren jonger dan 65 jaar worden verwezen naar de cardioloog voor nadere diagnostiek

**Kernaanbeveling 5:**

Bij patiënten ouder dan 65 jaar met paroxysmaal of chronisch atriumfibrilleren **zonder** hoog risico op TIA of CVA (afwezigheid van hypertensie (nu of in de voorgeschiedenis), diabetes mellitus, hartfalen, coronaire hartziekte of reumatische hartklepafwijking) wordt acetylsalicylzuur 80 mg per dag voorgeschreven. Bij patiënten ouder dan 65 jaar met paroxysmaal of chronisch atriumfibrilleren **met** een hoog risico op TIA of CVA (bij aanwezigheid van hypertensie (nu of in de voorgeschiedenis), diabetes mellitus, hartfalen, coronaire hartziekte of reumatische hartklepafwijking) wordt een coumarinederivaat voorgeschreven, tenzij hiervoor een contra-indicatie bestaat.

#### **3. Cardiovascular risk management/ Cardiovasculair risicomanagement** (M84; 2006)

**Kernaanbeveling 1:**

De huisarts brengt het cardiovasculair risicoprofiel in kaart bij:

- patiënten met hart- en vaatziekten (HVZ) of diabetes mellitus type 2 (DM2);
- patiënten die bekend zijn met een systolische bloeddruk ≥ 140 mmHg of totaal cholesterolgehalte ≥ 6,5 mmol/l;
- rokende mannen ≥ 50 jaar en rokende vrouwen ≥ 55 jaar;
- patiënten die reeds worden behandeld met antihypertensiva of statines.

Dit risicoprofiel bestaat uit de volgende factoren:

- leeftijd;
- geslacht;
- roken;
- systolische bloeddruk;
- lipidenspectrum (TC, HDL, TC/HDL-ratio, LDL, triglyceriden);
- glucosegehalte;
- familieanamnese (vader, moeder, broer of zus met HVZ <60e levensjaar);
- voeding (gebruik van verzadigd vet, vis, groente en fruit, zout);
- alcoholgebruik;
- lichamelijke activiteit;
- body mass index en middelomtrek

**Kernaanbeveling 2:**

Aan patiënten met HVZ, DM2 of een geschat 10-jaarsrisico van sterfte door HVZ ≥5% (met behulp van SCORE risicofunctie of risicometer) worden adviezen gegeven over voldoende bewegen, gezonde voeding, beperkt alcoholgebruik en, indien van toepassing, stoppen met roken.

**Kernaanbeveling 3:**

Aan patiënten met HVZ wordt acetylsalicylzuur 1dd 80 mg voorgeschreven tenzij er een indicatie is voor orale antistollingstherapie (bijvoorbeeld bij boezemfibrilleren of structurele hartafwijking). Tevens wordt behandeling met een cholesterolverlager (statine) geadviseerd, tenzij bij een LDL <2,5 mmol/l en geen sterk verhoogd risico (bijvoorbeeld recidiverend hartinfarct, sterk belaste familieanamnese of clustering van risicofactoren).

**Kernaanbeveling 4:**

Bij een geschat 10-jaarsrisico van sterfte door HVZ lager dan 5% is medicamenteuze behandeling van licht tot matig verhoogde bloeddruk (SBD 140-180 mmHg) en/of licht tot matig verhoogd cholesterolgehalte (TC/HDL-ratio 5-8) meestal niet zinvol.

**Kernaanbeveling 5:**

Indien besloten wordt tot antihypertensieve therapie bij patiënten zonder HVZ en zonder DM2, wordt geadviseerd te starten met een diureticum in een lage dosering.

**Kernaanbeveling 6:**

Bij medicamenteuze behandeling met cholesterolverlagers wordt geadviseerd te starten met simvastatine of pravastatine 40 mg. Bij patiënten met HVZ of DM2 waarbij de LDL-streefwaarde <2,5 mmol/l niet haalbaar is, kan bij patiënten met een sterk verhoogd risico (bijvoorbeeld recidiverend hartinfarct, sterk belaste familieanamnese of clustering van risicofactoren) worden overwogen om (initieel of in tweede instantie) atorvastatine of eventueel rosuvastatine voor te schrijven.

**Kernaanbeveling 7:**

Na instelling van de behandeling met antihypertensiva en/of cholesterolverlagers wordt ten minste jaarlijks de therapie geëvalueerd. Het controleschema wordt verder individueel opgesteld, afhankelijk van het risicoprofiel, de (co)morbiditeit en de persoonlijke wensen.

#### **4. Cerebrovascular accident / CVA** (M81; 2004)

**Kernaanbeveling 1:**

Patiënten met een CVA (acute neurologische uitvalsverschijnselen, zich uitend in verlamming van ledematen of gelaat, spraakstoornissen, of anderszins) worden op korte termijn verwezen voor opname op een *stroke-unit*, tenzij de uitvalsverschijnselen slechts gering van omvang zijn of spontaan al sterk verbeteren.

**Kernaanbeveling 2:**

Bij patiënten met een CVA die thuisblijven omdat de uitvalsverschijnselen spontaan sterk verbeteren, wordt een cardiovasculair risicoprofiel opgesteld (zie NHG-Standaard Cardiovasculair risicomanagement). Tevens wordt direct gestart met acetylsalicylzuur 1dd 160 mg gedurende twee weken, waarna de dosering wordt verlaagd naar 1dd 80 mg.

**Kernaanbeveling 3**:

Bij patiënten met een CVA die thuisblijven omdat de uitvalsverschijnselen spontaan sterk verbeteren, wordt het voorschrijven van bloeddrukverlagende medicatie onmiddellijk na het CVA afgeraden. Het is beter daar twee weken mee te wachten tot de patiënt klinisch stabiel is.

**Kernaanbeveling 4:**

Bij patiënten met een CVA die thuisblijven, draagt de huisarts zorg voor een spoedige start van de revalidatie - in een verpleeghuis (dagbehandeling), revalidatiecentrum of thuis - en periodieke evaluatie van het beloop en de behoefte aan zorg.

**Key recommendation 4: ENG**:

For patients with a Cerebrovascular accident who stay at home, the general practitioner takes care of a quick start of the rehabilitation process - in a nursing home (outpatients’ treatment), rehabilitation centre or at home - and takes care of a periodical evaluation of the course of process and the need for care.

#### **Kernaanbeveling 5:**

Ook in de chronische fase (d.w.z. als er geen verbetering meer te verwachten is) geeft de huisarts voorlichting aan patiënten met een CVA en hun centrale verzorgers met het accent op praktische informatie die kan bijdragen aan een zinvolle en bevredigende dagbesteding. Ook worden zij geattendeerd op activiteiten van de patiëntenvereniging zoals lotgenotencontacten, partnercontacten en voorlichtingsbijeenkomsten.

#### **5. Depressive disorder/ Depressieve stoornis (depressie)** (M44; 2003)

**Kernaanbeveling 1:**

De huisarts stelt de diagnose depressieve stoornis bij vijf van de volgende symptomen, waaronder ten minste één van de twee kernsymptomen***, gedurende ten minste twee weken:

- depressieve stemming gedurende het grootste deel van de dag, bijna elke dag*;
- duidelijke vermindering van interesse of plezier in alle of bijna alle activiteiten gedurende het grootste deel van de dag, bijna elke dag*;
- duidelijke gewichtsvermindering of gewichtstoename;
- slapeloosheid of overmatig slapen, bijna elke dag;
- psychomotorische agitatie of remming, bijna elke dag;
- moeheid of verlies van energie, bijna elke dag;
- gevoelens van waardeloosheid of buitensporige of onterechte schuldgevoelens, bijna elke dag;
- verminderd vermogen tot nadenken of concentratie of besluiteloosheid, bijna elke dag;
- terugkerende gedachten aan de dood, terugkerende suïcidegedachten zonder dat er specifieke plannen zijn gemaakt, of een suïcidepoging of een specifiek plan om suïcide te plegen

**Kernaanbeveling 2:**

Het beleid wordt vooral bepaald door de mate van de lijdensdruk en het disfunctioneren en afgestemd op de voorkeuren en wensen van de patiënt.

**Kernaanbeveling 3**:

Bij depressieve klachten, waarbij niet aan de criteria voor een depressieve stoormis wordt voldaan (zie kernaanbeveling 1), worden antidepressiva **niet** aangeraden omdat hiervan geen relevante effecten te verwachten zijn.

**Kernaanbeveling 4**:

Indien een behandeling met antidepressiva wordt gestart, kan de huisarts kiezen voor een tricyclisch antidepressivum (TCA) of een specifieke serotonineheropnameremmer (SSRI). De keuze wordt in overleg met de patiënt bepaald en hangt af van de aanwezigheid van contra-indicaties, co-morbiditeit, potentiële bijwerkingen en eerdere ervaringen.

#### **Kernaanbeveling 5:**

Na vier tot zes weken wordt het effect van de medicatie en eventuele bijwerkingen geëvalueerd. Bij voldoende respons en geen of acceptabele bijwerkingen wordt de medicatie in principe 6 maanden voortgezet.

**6. Eye Inflammation (‘Red eye’)/ Het rode oog** (M57; 2006)

**Kernaanbeveling 1:**

Bij een rood oog gepaard gaande met pijn, daling van het gezichtsvermogen of lichtschuwheid (indien niet veroorzaakt door keratoconjunctivitis fotoelectrica, corpus alienum of ander trauma), wordt de visus bepaald, de pupillen en pupilreacties beoordeeld en nader onderzoek van de cornea verricht met behulp van fluoresceïnekleuring.

**Kernaanbeveling 2:**

Bij diffuse roodheid en afwezigheid van jeuk, alarmsymptomen (pijn, visusdaling of lichtschuwheid) en cornea-afwijkingen, is er waarschijnlijk sprake van een infectieuze conjunctivitis. Indien de klachten korter dan drie dagen duren of er bestaat niet veel hinder, kan worden afgewacht zonder antibiotische behandeling.

**Kernaanbeveling 3:**

Als bij een (vermoedelijk) bacteriële conjunctivitis (diffuse roodheid,’s ochtends dichtgeplakte ogen en afwezigheid van jeuk, alarmsymptomen en cornea-afwijkingen) wordt besloten tot antibiotische behandeling, gaat de voorkeur uit naar chlooramfenicol oogzalf 1% 2-4 dd. Bij een blefaritis kan ook fusidinezuur worden voorgeschreven, in andere gevallen van conjunctivitis is dit middel niet zinvol gezien de resistentieontwikkeling.

**7. Rhinosinusitis/ Rhinosinusitis** (M33; 2005)

**Kernaanbeveling 1:**

Bij patiënten met rhinosinusitis geeft de huisarts voorlichting over het gunstige beloop van rhinosinusitis en adviezen over symptoombestrijding (stomen, (fysiologische) zoutoplossing via neusdruppels of spray, decongestiva, pijnstilling).

**Kernaanbeveling 2:**

Uitsluitend bij patiënten met een (verhoogd risico op een) afwijkend beloop wordt een antimicrobiële behandeling overwogen, d.w.z. bij:

- een patiënt die ernstig ziek is;
- alarmsymptomen (oedeem of roodheid van de oogleden van één oog, visusstoornissen zoals acuut verminderde visus of gestoorde volgbeweging, neurologische symptomen zoals meningeale prikkeling of uitvalsverschijnselen, suf of apathisch gedrag, en slecht drinken bij zuigelingen);
- opnieuw koorts na een aantal koortsvrije dagen binnen één klachtenepisode;
- klachten die in een periode van 2 weken niet afnemen;
- meer dan 3 klachtenepisodes per jaar;
- een gestoorde afweer (zoals slecht ingestelde diabetici, chronische corticosteroïdengebruikers, HIV-patiënten met een verlaagd aantal T-cellen, patiënten die een chemotherapeutische of radiotherapeutische behandeling ondergaan)

In deze gevallen wordt doxycycline of amoxicilline voorgeschreven gedurende één week. Bij kinderen jonger dan 13 jaar, bij zwangeren en bij vrouwen die borstvoeding geven is amoxicilline het middel van eerste keus. Bij allergie of intolerantie kan de huisarts kiezen voor erytromycine of azitromycine.

**8. Sexually transmissible diseases/ Het soa-consult** (M82; 2004)

**Kernaanbeveling 1:**

Volg bij patiënten zonder klachten maar met ongerustheid na seksueel contact, in overleg met de patiënt, het volgende onderzoeksbeleid:

- bij heteroseksuele jongeren: onderzoek chlamydia en gonorroe.
- bij wisselende heteroseksuele contacten: onderzoek chlamydia en gonorroe en bij grote ongerustheid tevens syfilis, hepatitis B, HIV-infectie.
- bij homo- of biseksuele contacten, homo- of biseksuele partner, of als patiënt of diens partner werkzaam is in prostitutie of prostituant is: onderzoek chlamydia, gonorroe, syfilis, hepatitis B en HIV-infectie.
- als partner intraveneuze drugsgebruiker is: onderzoek hepatitis B en HIV-infectie.
- als patiënt en/of partner afkomstig zijn uit HIV-endemische gebieden: onderzoek chlamydia, gonorroe, syfilis, hepatitis B en HIV-infectie.
- als patiënt en/of partner afkomstig zijn uit hepatitis-B-virus-endemische gebieden: onderzoek hepatitis B.

**Kernaanbeveling 2:**

Verricht bij mannen met een urethritis (afscheiding uit de penis of pijn in de urethra met leukocyturie (>10 leukocyten per gezichtsveld in het sediment van de eerstestraalsurine)) onderzoek naar een chlamydia-infectie en gonorroe en start direct met (eenmalig) 1 gram azitromycine oraal. Geef bij een grote kans op gonorroe (purulente afscheiding, klachten enkele dagen na contact waarbij besmetting zou hebben plaatsgevonden, prostitutie, homo- of biseksueel contact), of wanneer de kans dat er geen vervolgcontact zal zijn groot is, eenmalig 1 gram azitromycine oraal plus eenmalig 1 gram cefotaxim intramusculair.

**Kernaanbeveling 3**:

Chlamydiadiagnostiek bij een vrouw wordt verricht door afname van materiaal voor PCR uit cervix en urethra. Voor het uitsluiten van Chlamydia bij een vrouw **zonder** klachten is een PCR op urine of een door de vrouw zelf afgenomen uitstrijk met een vaginale wat (indien beschikbaar) ook voldoende.

**Kernaanbeveling 4**:

De huisarts motiveert patiënten met een chlamydia-infectie, gonorroe of acute hepatitis B om de seksuele partner(s) tot een half jaar terug te waarschuwen en op te sporen. Bij dragerschap van het hepatitus-B-virus moeten alle seksuele partners en gezinsleden worden gewaarschuwd. Als de patient HIV-positief is, is het zinvol zo mogelijk alle seksuele partners uit het verleden op te sporen, eventueel vanaf het moment dat een eerdere test negatief was. Als de patiënt het moeilijk vindt om contact op te nemen, kan de huisarts hiervoor schriftelijk materiaal (een zogenaamde waarschuwingsstrook van de Stichting Soa-Aids-Nederland) meegeven of de GGD inschakelen voor contactopsporing, bijvoorbeeld bij anonieme contacten.

**9. Sleeping disorder/ Slaapproblemen en slaapmiddelen** (M23; 2005)

**Kernaanbeveling 1:**

Bij slapeloosheid vindt aanvullend onderzoek alleen plaats op grond van ervaren klachten. Bij het *restless legs* syndroom wordt het Hb en MCV bepaald.

**Kernaanbeveling 2:**

De huisarts geeft voorlichting over wat onder normale slaap wordt verstaan en probeert te zorgen voor een meer relativerende opstelling van de patiënt ten opzichte van een korte slaap.

**Kernaanbeveling 3**:

Bij ‘gewone’ slapeloosheid worden slaapadviezen of ontspanningsoefeningen gegeven en is slaapmedicatie niet nodig.

**Kernaanbeveling 4**:

Slaapmedicatie wordt overwogen bij acute psychosociale problemen, bij passagère verstoring van het dag-/nachtritme, zoals bij een jet lag, en bij chronische somatische aandoeningen met aanhoudende klachten ondanks specifieke therapie.

**Kernaanbeveling 5**:

Indien men kiest voor slaapmedicatie, schrijft men een kortwerkend middel voor, zoals temazepam 10-20 mg (ouderen 10 mg) of zolpidem 5-10 mg (ouderen 5 mg), niet meer dan 5 tot 10 tabletten, met als gebruik zo nodig of intermitterend. Dagelijks gebruik moet worden vermeden.

**Kernaanbeveling 6**:

Bij chronisch slaapmiddelengebruik probeert men de patiënt te laten stoppen via een minimale interventiestrategie (met behulp van stopbrieven, beschikbaar via NHG-Patiëntenbrieven).

**Kernaanbeveling 7**:

Bij slaapapneu, narcolepsie en een ernstige vorm van het vertraagde slaapfasesyndroom (te laat afgestemde biologische klok) dient men de patiënt door te verwijzen voor nadere diagnostiek en/of behandeling.

#### **10. Thyroid disorder/ Schildklieraandoeningen** (M31; 2006)

**Kernaanbeveling 1:**

Streef bij de behandeling van een patiënt met **hypothyreoïdie** naar een normale TSH-waarde en stel de medicatie (met levothyroxine) verder bij op grond van klachten. Spreek in de instelfase, niet eerder dan zes weken na de laatste doseringsverandering, een laboratoriumcontrole (TSH en vrij T4) af. In het eerste jaar nadat de patiënt klachtenvrij is geworden en het TSH en vrije T4 door medicatie zijn genormaliseerd, vinden de controles elke drie maanden plaats. Spreek vervolgens een jaarlijkse controle af, levenslang.

**Kernaanbeveling 2:**

Indien de huisarts specifieke kennis heeft van schildklieraandoeningen kan een patiënt met **hyperthyreoïdie** (ziekte van Graves) door de huisarts medicamenteus worden behandeld via de combinatiemethode. Hierbij wordt de schildklier eerst volledig stilgelegd met een thyreostaticum (bij voorkeur thiamazol 1dd30 mg), waarna levothyroxine wordt bijgegeven. Bespreek de voor- en nadelen van de verschillende behandelopties (medicamenteus, radioactief jodium, chirurgie) met de patiënt en betrek hem of haar bij de besluitvorming.

**Kernaanbeveling 3**:

De huisarts verwijst patiënten met een solitaire nodus of met een dominante nodus in een multinodulair struma naar een internist voor aanvullende diagnos­tiek.

**11. Transient Ischemic Attack/ TIA** (M45; 2004)

**Kernaanbeveling 1:**

De huisarts stelt de diagnose *transient ischaemic attack* (TIA) als:

- de uitvalsverschijnselen acuut en zonder voortekenen waren begonnen en zich binnen 5 minuten volledig ontwikkeld hadden;
- alle verschijnselen tegelijk ontstonden en de uitval langer dan 1 minuut duurde;
- de uitvalsverschijnselen niet meer bij lichamelijk onderzoek aanwezig zijn;
- de uitvalsverschijnselen te verklaren zijn vanuit een stoornis in de bloedvoorziening van de hersenen door de linker of rechter a. carotis interna of de a. basilaris (zie tabel).

**Tabel: Uitvalsverschijnselen die passen bij een TIA**

| **Lokalisatie stoornis in bloedvoorziening** | **Neurologisch begrip** | **Verschijnselen** |
| --- | --- | --- |
| Arteria carotis interna sinistra* | Hemiparese | totale of gedeeltelijke verlamming of vaardigheidsstoornis van de rechter bovenste extremiteit, rechter onderste extremiteit en het rechter aangezicht |
|  | Sensibiliteitsstoornis^†^ | doof, verminderd of tintelend gevoel van de rechter arm, het rechter been of het rechter aangezicht |
|  | Homonyme hemianopsie^‡^ | zwart of niets zien met beide ogen in de rechter helft van het gezichtsveld |
|  | Afasie | niet op de goede woorden komen, gesproken taal niet begrijpen, de woorden wel weten, maar deze niet kunnen uitspreken of vreemde woorden zeggen |
|  | Dysartrie^‡^ | onduidelijke spraak |
|  | Amaurosis fugax | zwart of niets zien met het linker oog. Soms betreft de uitval alleen het onderste of bovenste deel van het gezichtsveld. Er zijn gewoonlijk geen andere uitvalsverschijnselen. |
| Arteria basilaris | Parese | totale of gedeeltelijke verlamming of vaardigheidsstoornis die zich niet per se beperkt tot een lichaamshelft |
|  | Sensibiliteitsstoornis | doof, verminderd of tintelend gevoel in linker of rechter lichaamshelft of beiderzijds |
|  | Hemianopsie^‡^ | zwart of niets zien met beide ogen in de linker of de rechter helft van het gezichtsveld |
|  | Combinaties van:^†^ |  |
|  | - vertigo | draaiduizeligheid |
|  | - dysartrie^‡^ | onduidelijk spreken |
|  | - diplopie | boven of naast elkaar zien van twee gelijke beelden, hetgeen verdwijnt als een van de ogen wordt gesloten |
|  | - dysfagie | verslikken |
|  | - ataxie | stuurloosheid of zwalken (dronkemansgang) |

* Een stoornis in de bloedvoorziening door de arteria carotis interna dextra gaat gepaard met dezelfde uitvalsverschijnselen, maar dan aan de andere kant. Afasie treedt dan echter alleen op als het spraakcentrum in de rechter hersenhelft is gelokaliseerd, hetgeen doorgaans het geval is bij linkshandigen.

^†^ Indien deze verschijnselen geïsoleerd voorkomen, mag de diagnose TIA niet gesteld worden.

^‡^ Op basis van een hemianopsie of dysartrie zonder andere uitvalsverschijnselen valt niet uit te maken of de stoornis in de cerebrale bloedvoorziening in de arteria basilaris of een van de carotiden gelokaliseerd is.

**Kernaanbeveling 2:**

Bij patiënten met een TIA wordt een cardiovasculair risicoprofiel opgesteld (zie NHG-Standaard Cardiovasculair risicomanagement). Tevens wordt gestart met acetylsalicylzuur 1dd 160 mg ineens, waarna de behandeling (levenslang) wordt voortgezet met 1dd 80 mg. Daarnaast komen zij in aanmerking voor (2 x daags 200 mg) dipyridamol met gereguleerde afgifte (zie addendum). Bij atriumfibrilleren heeft het gebruik van een coumarinederivaat de voorkeur boven acetylsalicylzuur. Bij contra-indicaties voor acetylsalicylzuur is clopidogrel een goed alternatief.

**Kernaanbeveling 3:**

Patiënten met voorbijgaande uitvalsverschijnselen die wijzen op een stoornis in het stroomgebied van de arteria carotis interna (zie tabel), worden binnen drie dagen verwezen naar de neuroloog voor een duplexscan (combinatie van echografie en doppleronderzoek), om te beoordelen of er een indicatie bestaat voor carotis-chirurgie.

#### **12. Urinary Tract Infections/ Urineweginfectie** (M05; 2005)

#### **Kernaanbeveling 1:**

#### Het urineonderzoek bij klinische verdenking op urineweginfecties bestaat in eerste instantie uit een nitriettest, waarna bij een negatieve uitslag een dipslide wordt ingezet.

#### **Kernaanbeveling 2:**

#### Ongecompliceerde urineweginfecties, d.w.z. urineweginfecties bij niet-zwangere, overigens gezonde vrouwen, dienen in eerste instantie behandeld te worden met nitrofurantoïne. Bij overgevoeligheid voor nitrofurantoïne wordt trimethoprim geadviseerd.

**Kernaanbeveling 3**:

Bij een gecompliceerde urineweginfectie, d.w.z bij tekenen van weefselinvasie (koorts, rillingen, algemeen ziek-zijn, flank- of perineumpijn) of urineweginfectie bij patiënten uit een risicogroep (mannen, zwangere vrouwen, personen jonger dan 12 jaar, patiënten met afwijkingen aan de nieren of urinewegen in de voorgeschiedenis (zoals ernstige nierinsufficiëntie, cystennieren, nierstenen, een neurogene blaas of bemoeilijkte mictie), patiënten met een verminderde weerstand (zoals tgv. bestraling, immunosuppressiva of diabetes mellitus), patiënten met een verblijfskatheter) dient voorafgaand aan de behandeling urine te worden verzameld voor kweek en resistentiebepaling.

**Kernaanbeveling 4**:

Bij een gecompliceerde urineweginfectie dienen patiënten **met** tekenen van weefselinvasie, evenals alle jongens tot 12 jaar, meisjes tot en met 4 jaar en daarnaast patiënten met aandoeningen van de nieren of urinewegen, een verminderde weerstand (m.u.v. diabeten) of een verblijfskatheter, gedurende 10 dagen te worden behandeld met amoxicilline/ clavulaanzuur, totdat de uitslag van de kweek en resistentiebepaling bekend is. Bij overgevoeligheid dient dit te worden vervangen door co-trimoxazol of een fluorchinolon (norfloxacine 2dd400 mg of ciprofloxacine 2dd500 mg, maar niet in de zwangerschap of tijdens lactatie of bij leeftijd < 16 jaar).

#### **Kernaanbeveling 5:**

#### Bij een gecompliceerde urineweginfectie bij patiënten **zonder** tekenen van weefselinvasie dienen mannen, zwangeren, meisjes van 5-12 jaar en diabeten gedurende 7 dagen te worden behandeld met nitrofurantoïne totdat de uitslag van de kweek en resistentiebepaling bekend is. Bij overgevoeligheid voor nitrofurantoïne wordt gekozen voor 7 dagen trimethoprim.
